# Haploid Induction in *Indica* Rice: Exploring New Opportunities

**DOI:** 10.3390/plants12173118

**Published:** 2023-08-30

**Authors:** Ruwani Mayakaduwa, Tara Silva

**Affiliations:** Department of Plant Sciences, University of Colombo, Colombo 00300, Sri Lanka; ruwani.mayakaduwa@pts.cmb.ac.lk

**Keywords:** *indica* rice, doubled haploids, androgenesis, haploid inducer lines, genome editing

## Abstract

Haploid plants are of significant interest to crop breeders due to their ability to expedite the development of inbred lines. Chromosome-doubling of haploids, produced by either in vitro or in vivo methods, results in fully homozygous doubled haploids. For nearly five decades, in vitro methods of anther and microspore culture have been attempted in many crops. In rice, in vitro methods are used with some success in *japonica* cultivars, although *indica* types have remained recalcitrant to a large extent. This review aims to explore the reasons for the lack of success of in vitro methods in *indica* rice and discuss new advancements in in vivo haploid induction protocols in other cereals and their relevance to rice. In particular, the current level of understanding of in vivo haploid inducer systems that utilize *MTL* and *CENH3* mutants is analyzed in detail. One notable advantage of in vivo haploid induction systems is that they do not require tissue culture competence. This makes these methods more accessible and potentially transformative for research, offering a pragmatic approach to improving *indica* rice cultivars. By embracing these in vivo methods and harnessing the power of gene editing technologies like CRISPR/Cas9 systems, breeders can reshape their approach to *indica* rice improvement.

## 1. Introduction

Cultivated rice, *Oryza sativa* L., consists of two main subspecies, *indica* and *japonica* [1]. *Indica* rice is grown extensively in tropical and subtropical Asia, regions that have the highest population density in the world, with China and India accounting for over 50% of global rice production [2]. *Indica* rice is the staple food of more than half the world’s population and thus is the main contender for fulfilling the sustainable development goal of zero hunger [3]. The demand for rice is expected to grow further, with the global population projected to reach 10.6 billion by 2050 [4].

Although much progress has been made in the genetic improvement of *indica* rice and an array of superb cultivars have been released, vigorous breeding efforts need to continue in order to meet the future challenges of population growth and climate change. Conventional breeding strategies of hybridization and selection need to be augmented by the utilization of new breeding approaches, including marker-assisted selection, genetic modification, and gene editing, with a renewed focus on the development of new cultivars suitable to withstand biotic stresses caused by numerous rice diseases [5] and environmental stresses caused by drought, flooding, or saltwater intrusion [6].

Rice is a short-duration crop of 3–4 months, with usually two cropping cycles per year. It takes six or more years to release a new cultivar through conventional breeding, as inbreeding has to be repeated over several years to achieve purebred lines from an initial cross. Haploids are of major interest to plant breeders because, by doubling their chromosome number, fully homozygous doubled haploids can be produced. The conversion of haploids to doubled haploids ensures fixing homozygosity more rapidly than conventional methods of line development such as selfing and backcrossing [7,8,9,10].

The natural occurrence of haploids was first reported in the weed *Datura stramonium* [11] and has since been observed in other plant species [7,12]. However, the practical value of such natural spontaneous haploids or doubled haploids is limited as they occur at low frequencies [13]. Therefore, for commercial plant breeding, haploid induction has to be made more efficient. Both in vitro and in vivo systems can be employed for the production of haploids. While in vivo methods have proven to be more efficient than in vitro techniques for haploid induction in crops such as maize [14], a reliable in vivo method is not known for many important crops, including rice. On the other hand, in vitro culture of haploid cells has the potential to be applied to many plant species, even though success will vary among different groups and genotypes.

In this review, we examine the current status of research with regard to the application of in vitro techniques for rice haploid induction and discuss the problems that the *indica* types are faced with. We also highlight the recent advances in in vivo approaches for haploid induction in rice. Especially with regard to the in vivo methods, the progress made in other cereals is analyzed, and the transferability of these developments to rice is examined, particularly in view of the availability of new tools for gene editing such as the CRISPR/Cas9 system.

## 2. In Vitro Techniques for Induction of Haploids and Doubled Haploids

Both maternal and paternal haploids have been produced in vitro through gynogenesis and androgenesis, respectively. In gynogenesis, haploids are induced from the female gametophyte, whereas in androgenesis, haploid plants are produced from male gametes. Gynogenesis is used less frequently in commercial applications, except in a few crops such as onion and sugar beet. This is mainly because ovules have a limited number of haploid cells (compared to the large number of pollen grains in an anther), which reduces the probability of success in inducing haploids [7,8].

Androgenesis was first successfully attempted in *Datura innoxia,* where haploid embryos were induced from anthers grown in vitro on Nitsch’s or White’s basal nutrient media [15]. Rapeseed, tobacco, wheat, and barley are a few of the important crops that have been successfully improved using androgenesis.

However, even after several decades of experimentation, the application of doubled haploid technology is still limited to only a few species due to biological and technical barriers. Furthermore, laboratory protocols that have been developed for in vitro induction of haploids and doubled haploids vary among species and even within a species [8].

### 2.1. Microspore-Derived Doubled Haploids in Rice Breeding: Standard Protocol

**Figure 1:**
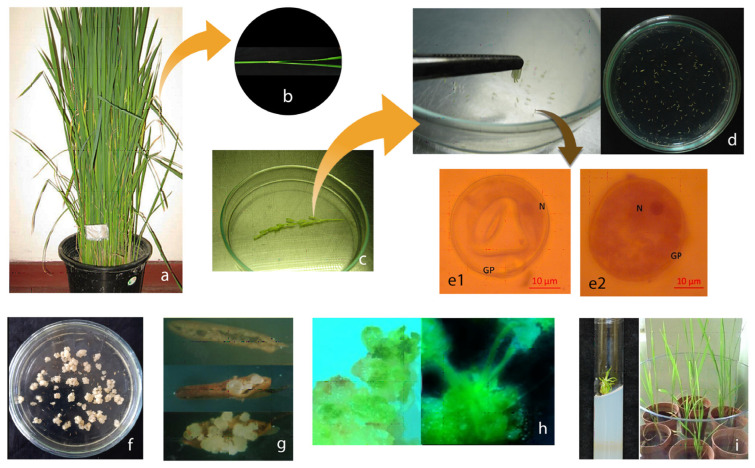
Main steps in the standard in vitro protocol to develop doubled haploids in *indica* rice through anther culture. (**a**) Anther donor plants grown under standard agricultural practices (**b**) A panicle at booting maturity (**c**) Cold-stressed spikelets following surface disinfection (**d**) Fresh anthers cultured on callus induction medium (**e1**,**e2**) Microspores at uni-nucleate stage; N = nucleus; GP = germ pore; *scale bar* = *10 µm* (**f**) Calluses induced on stimulated anthers (**g**) Growth of calluses on split-opened anthers (**h**) Green shoot formation in anther-derived calluses (**i**) Green plantlets regenerated.

While androgenic haploids can be induced from in vitro cultured anthers or isolated microspores, for cereals, including rice, anther culture is preferred due to its convenience. In rice, stimulation of the anthers often leads to callus formation initially. Therefore, regeneration of haploids or doubled haploids occurs through a callus phase rather than via direct organogenesis. In rice, the stage of microspore maturity is crucial for re-directing its development pathway in vitro. Usually, panicles at the booting stage carry responsive microspores of the mid- to late uni-nucleate or early bi-nucleate stage that are suitable for in vitro culture [16,17,18,19]. Surface disinfected panicle boots are subjected to cold stress (at 10 °C for 7–10 days) to convert microspores from the gametophytic to the sporophytic pathway of development. Callus induction and regeneration are regulated by specific plant growth regulators and incubation conditions [10,18,19,20,21]. The regenerated plants may be haploid, in which case they are chromosome-doubled, or they may have undergone spontaneous doubling of chromosomes in vitro to yield doubled haploids that are fully homozygous [7,22,23]. Figure 1 illustrates the step-wise process for developing androgenic regenerants in *indica* rice.

### 2.2. In Vitro Haploid Induction in Rice: The Current Scenario

Producing varieties with superior yield, good grain quality, and resistance to abiotic and biotic stresses are some important targets of rice breeding programs [24]. Moreover, fortification of the rice seed with extra nutrients to help overcome malnutrition among rice-consuming populations is a current focus. The development of doubled haploids through anther culture has sped up bio-fortification programs and accelerated the release of elite lines. Through anther culture, a number of superior rice varieties and some improved breeding lines, especially of the *japonica* type, were released in China, Korea, Japan, and the United States [25,26,27]. Several good characters from different *japonica* parents were combined in a single rice variety, “Hua Han Zhao” through multiple crosses followed by anther culture [28]. Few other rice varieties developed using anther culture-assisted breeding, such as “Xin Xu” and “Huayu 15” have been introduced as commercial cultivars [29]. Anther culture, integrated with molecular marker-assisted selection, has enabled the development of versatile breeding resources, including cytoplasmic male sterile maintainer lines [30].

In contrast to *japonica*, *indica* rice is widely recognized as recalcitrant for anther culture. *Indica* types suffer from poor callus induction and morphogenesis during callus induction and regeneration [9,19,20,26,31,32,33]. Low recovery of doubled-haploids has made androgenic applications less efficient for *indica* rice [34]. Successful adoption of androgenesis further depends on deploying effective methods for increasing the ratio of green to albino plant regeneration [9], converting haploids to doubled-haploids by applying anti-microtubular agents, and eliminating undesirable heterozygous diploids [35]. One of the success stories in *indica* rice anther culture is the release of the salt-tolerant variety “PSBRc50 Bicol” from a cross between two *indica* breeding lines (IR 5657-33-2 × IR 4630-22-2-5-1-3). While there are some sporadic reports of successful development of new *indica* varieties through anther culture [36,37], the technique is not used for regular breeding of *indica* rice. Therefore, a detailed analysis of the factors leading to recalcitrance and subsequent failure of *indica* rice anther culture is a timely requirement.

### 2.3. Factors Influencing Androgenic Success: Hereditary Factors

#### 2.3.1. The Genotype

The genotypic effect is a main factor determining androgenic success [38]. Large variability in the androgenic response has been observed between the two main subspecies (*japonica* and *indica*) of Asian cultivated rice. For example, when a high callus induction rate (41.9%) was reported in a *japonica* variety (‘Taipei 309’), the response was 0% in an *indica* variety (‘Suweon 290’) [39]. There is also wide variability in anther response among genotypes within a subspecies; in 7 inbred parents and 12 F1 hybrids of *indica* origin, callus induction frequencies varied from 3.6% to 51.7%, and green plant regeneration frequencies ranged from 1.6% to 82.9% [31]. Generally, genotypes that are better responsive for callus induction fail during morphogenesis due to the low heritability associated with genes controlling regeneration. Therefore, these two events must be equally developed in a successful anther culture system [31,40].

Studies on the heritability of androgenic traits suggest that over 90% of the variability is due to genetic effects [31]. Therefore, intersubspecific hybridization was able to resolve the genetic recalcitrance of *indica* rice to some extent [28,31,41], with pollen of F1 hybrids from *indica* × *japonica* crosses being more successful in callus induction and plant regeneration than pure *indica* parents [42].

A major problem associated with in vitro haploid induction in rice is the regeneration of albino shoots, which is also common in other cereals. The high frequency of albino production is an obstacle to the utilization of this technique [9]. Albinism is highly genotype-dependent, and in rice, *indica* cultivars are more prone to this problem than *japonica* types [40]. During normal gametogenesis, proplastids contained in microspores either convert to amyloplasts or eventually degenerate. In barley, it has been identified that genotypes showing a delayed transition of proplastids to amyloplasts tend to regenerate green shoots when plastids develop into chloroplasts under in vitro culture conditions [43].Albino plant production in rice has also been attributed to deletions in the plastid genome [44].Therefore, a better understanding of rice plastomes and chloroplast biogenesis is necessary to address the problem of albinism in rice.

#### 2.3.2. Microspore Maturity

The development stage of microspores is a crucial factor determining the success of anther culture [13,40,45]. During normal gametophytic development, haploid microspores first undergo an asymmetric nuclear division to form two distinct nuclei, vegetative and generative, producing bi-nucleate stage pollen. The generative nucleus divides again, forming two sperm nuclei in the tri-nucleated pollen grain. This serves as the functional unit to deliver the male gametes during fertilization [46]. For androgenesis, gametophytic development in pollen must be suppressed and switched to a sportophytic development mode. The re-programming can be induced only in immature pollen of a particular developmental stage [13,47]. In rice anther culture, microspores around the first mitotic division, which are at uni-nucleate (mid to late) to early bi-nucleate stages, were found to be the most responsive for sporophytic induction [16,17,18,19,48]. Therefore, accurate identification of pollen maturity is necessary prior to the culture of anthers. Pollen staging requires nuclear staining followed by cytological observation. Given that cytological observations are time-consuming, easily observable morphological markers that correlate well with microspore development are used in rice anther culture to correctly identify pollen maturity prior to culture [48]. The external morphological trait that is frequently used with rice anther culture is the internode distance between the flag and the penultimate leaf of the panicle boot [18,21,48,49,50,51]. Panicle length at the time of harvest, as well as the length ratio between anther and spikelet, have also been used as guides to determine microspore maturity [17,52,53]. In developing such guides for rice, adequate sampling of anthers should be carried out, representing the entire inflorescence, since maturity of spikelets varies along the different positions in the inflorescence. For best results, these markers need to be customized on an individual genotypic basis [40].

### 2.4. Factors Influencing Androgenic Success: Environmental Factors

#### 2.4.1. Donor Plant Environment

Growing conditions of anther donor plants have a major effect on in vitro anther response, so even the best responsive genotypes may fail due to changes in the growing environment [54]. The frequency of embryogenic pollen grains (P-grains) was reported to be directly regulated by the growth conditions of the donor plants [55]. Increased P-grain frequency in tobacco was possible by shifting the environmental conditions to short days and low temperatures. Light intensity, photoperiod, and the temperature under which the donor plants are maintained have been identified as critical factors determining androgenic success. In the *indica* rice cultivar, IR-43, the best anther culture efficiency was reported from donor plants that reached the booting stage under long days (>12 h), high solar radiation (>18 MJ m^−2^), sunshine (>8 h), and day/night temperatures (34 °C/24 °C) [56].

While specific growing conditions of donor plants have a bearing on the repeatability of anther culture success, growing plants under strict controlled environments has also not yielded the best results. In rice, anthers from field-grown plants produced a better response during in vitro culture compared to anthers harvested from plants grown under controlled conditions such as greenhouses or pots [56]. Also in wheat, anthers collected from greenhouse-grown plants were only half as responsive as the anthers harvested from field-grown plants [57]. Similar observations have been reported with other crops as well [54]. However, for temperate crops, it may not be practical to grow anther donor plants under natural field conditions. In fact, for several cereal crops, including triticale, barley, and rye, the optimal conditions for growing donor plants have been recommended in order to stabilize the in vitro response [57,58,59].

The embryogenic competence of pollen can be correlated with the donor plant’s physiology. Nutritional conditions of anther tissue and endogenous growth regulator levels are strongly regulated by the physiology of the plant [13,60]. It was also noted that the donor plant’s age is strongly linked with its physiological processes, and anthers from first-formed flowers or inflorescences were more competent during in vitro culture [60,61,62].

On the other hand, stressing the anther donor plants has also been useful to elicit a good anther response in some crops. For example, nutrient starvation, particularly of nitrogen and sugar [60], and off-season planting [63] were reported to enhance anther response. Increased anther culture efficiency has been reported by stressing rice plants by ratooning, during which the P-pollen grain population increased by twofold compared to those from the normal growth cycle [64]. Therefore, with certain species and specific genotypes, selective stress conditions given to donor plants can be useful to optimize the subsequent in vitro anther response.

#### 2.4.2. Anther Pre-Treatment

In almost all plant species that have been investigated, a stress pre-treatment of anthers or pollen was found to be essential to trigger an androgenic response [13,60,65]. Pre-treatment could be imposed either physically or chemically for embryogenic induction of microspores and can be applied to intact or excised inflorescences, excised anthers, or even to anther donor plants [66]. A wide range of stresses have been attempted on a number of crops [67]. Among different stresses that promote androgenesis, low-temperature pre-treatment is commonly applied for rice due to its proven efficacy [18,21,22,23,24,25,26,27,28,29,30,31,32,33,68]. Stress treatments such as osmotic shock and nutrient starvation have also been widely used with rice for the induction of calluses with high morphogenetic competence [22,69]. Other pre-treatments applied to different species include desiccation, centrifugation, γ-irradiation, use of microtubule disruptive agents, and exogenous application of growth regulatory compounds [65].

The effect of cold temperature pre-treatment on the in vitro androgenic response in rice has been analyzed in detail. It has been opined that low temperatures cause delayed development, encouraging more microspores to undergo reprogramming [70]. Cytoplasmic attenuation and mitochondrial condensation that result during cold stress were suggested to disturb the usual pollen germination process [71]. Disruption of pollen-tapetal attachment in cold-stressed anthers that caused nutrient starvation of pollen was also suggested as being important for androgenic induction [67,72,73]. Cold-stressed plants were shown to produce distorted or sterile pollen that had lost their usual gametophytic ability [74]. During rice pollen development, the period of highest cold sensitivity was observed to coincide with the best period of tapetal activity, which is during the transition from tetrads to the early uni-nucleate microspore stage [75].

Physiological studies of highly regenerable callus cells induced under osmotic stress conditions revealed that their fresh weight, water content, cellular water, and osmotic potential decreased [76]. The osmotic treatment-led cellular mechanisms were explained on the basis of plasmolysis, which disrupts cellular interconnections in cell clusters. Therefore, more cells could be physiologically isolated and triggered to develop independently into adventive embryos [77].

Cytoplasmic and nuclear changes have been observed to occur in pollen grains during nutrient starvation. Some of the changes are: dedifferentiation of plastids, dilation of the generative cell wall, presence of a large vacuole, loss of nuclear pores in the vegetative nucleus, structural changes in chromatin and nucleolus, and size reduction of the nucleolus [78]. Specific phospho-proteins were also observed to have related functions during embryogenic induction [79]. The starvation of pollen either by removing sugar or nitrogen from the in vitro environment resulted in the efficient formation of P-grains carrying inherent induction potential [80]. Starvation has been identified as a key determinant leading to a series of degradation events in the pollen grains that are essentially required for the androgenic switch.

Even though some emphasis has been given to cytological changes in the microspores during pre-treatments and early in vitro development [46,65,66,81], investigations on cellular mechanisms and histological changes during successful morphogenesis are rare, particularly for microspore-derived sporophytic development in rice [82].

#### 2.4.3. In Vitro Culture Conditions

While the overriding factor determining androgenic success may be genetic, the androgenic potential of highly recalcitrant genotypes, particularly *indica* subspecies, has been optimized by micromanipulating the composition and conditions of the in vitro culture environment [20]. Generally, the most widely used basal nutrient media for improving anther culture in cereals, particularly in *indica* rice, are N_6_ [83] and MS [84]. While each genotype may demand a delicately balanced nutritional regime during different phases of in vitro growth [85], it is equally important to maintain a correct balance of plant growth substances in the culture medium. Culture media not only supply the tissues with nutrients but also determine the direction of development. In vitro morphogenesis is regulated largely by the balance between auxins and cytokinins [33,49,86].

Conditions under which cultures are incubated can also affect androgenic success [22,60]. It is important that culture composition and conditions are optimized in a way that ensures not only the improvement of callus induction potential but also the embryogenic potential of induced calluses.

### 2.5. Plants Regenerated through In Vitro Haploid Cell Culture

The primary objective of the anther or microspore culture method is to obtain fully homozygous diploid plants through either induced or spontaneous doubling of the haploid chromosome complement. However, it has been noted that a mixture of different ploidy levels can occur in plants regenerated from in vitro culture. In certain *indica* rice cultivars, 50% or more of anther-derived plants were reported to be diploid and a considerable proportion haploid, while a few mixoploids or tetraploids were also observed [23,87]. Others have reported a spontaneous chromosome doubling rate of 30–40% for rice [88]. While the spontaneous chromosome doubling rate is high in rice, anther culture efficiency can be improved further by treating the regenerated haploid plants with chromosome doubling agents such as colchicine and oryzalin [88].

Spontaneous doubling of chromosomes can occur in cells through endomitosis—chromosome duplication without nuclear division, in which case the diploids will be fully homozygous. However, in anther culture, there is the possibility of regeneration of shoots from somatic cells of anther tissue. Therefore, it is necessary to evaluate diploid plants that are regenerated through anther culture for their origin, whether gametic (from microspores) or somatic (from cells of the anther wall). This is usually examined using molecular markers. Simple Sequence Repeats (SSRs), which are abundant and well-distributed throughout the rice genome, have been the marker of choice for establishing the homozygosity of anther-derived plants [9,53].

## 3. In Vivo Methods of Haploid Induction

### 3.1. Wide Hybridization and Embryo Rescue

Alongside in vitro methods such as anther and microspore culture for haploid induction, wide hybridization is utilized as a technique for generating haploid plants in cereal crops. In some wide crosses, although fertilization occurs between gametes of two different species, resulting in a viable embryo, there is often the elimination of chromosomes from one genome in the developing embryo due to the incompatibility of the combined genomes. The ‘bulbosum’ method was the first such technique to be developed in cereals. In this method, cultivated barley (*Hordeum vulgare*) lines are crossed with *H. bulbosum*. Soon after fertilization, there is a unilateral loss of *H. bulbosum* chromosomes from the dividing cells of the hybrid embryo. Eventually all of the *H. bulbosum* chromosomes are eliminated, and viable embryos can be recovered through embryo rescue to produce haploid plants of *H. vulgare*. Interspecific hybridization followed by embryo rescue in barley was first described as a method of haploid induction by Kasha and Kao in 1970 and has since been utilized as a reliable technique to produce haploids of *H. vulgare* [89]. While the efficiency of haploid induction depends on genetic factors and environmental conditions (chromosome elimination occurs more efficiently at temperatures over 18 °C), it is a robust technique that is able to generate haploid embryos in up to 30% of the florets pollinated [90].

Wide hybridization has been used to generate haploids in other cereals too. For example, polyhaploids of hexaploid wheat, *T. aestivum*, have been produced by crossing wheat with other grasses such as teosinte, maize, sorghum, and pearl millet [91,92]. Widely used wheat × maize systems and oat × maize crosses for haploid induction in wheat and oat, respectively, have shown that maize is the commonly used pollen donor for generation of haploids through intergeneric hybridization in cereals other than barley [93,94]. In the maize system, chromosomes of the pollen parent are lost preferentially from the developing embryo, allowing the recovery of haploid plants from the female parent [92]. Since these early studies, maize-mediated in vivohaploid systems have been extensively used as a practical breeding tool to generate haploids in several crop species [95]. In contrast toanther and microspore culture, this technique is not genotypically dependent. Furthermore, haploids generated through wide crossing do not suffer from albinism.

While wide hybridization has worked reasonably well for haploid induction in crops such as barley, maize, and wheat, the technique is not applicable for rice since wide hybridization in the genus *Oryza* has not yielded haploids so far. Intergeneric crosses of rice with maize and pearl millet were attempted but without success [96]. Interspecific crosses of *O. sativa* with 22 wild species of *Oryza* have not been evaluated properly for their haploid induction potential.

Despite wide hybridization being used as a practical tool for haploid induction in several plant species, until recently, the actual mechanism of chromosome elimination was poorly understood. While centromere incompatibility and asynchrony between cell cycles were previously suggested to account for genome elimination in wide crosses [97,98], in the recent past, interest has been directed towards the role played by CENH3, a centromere-specific histone, on chromosome segregation during nuclear division. New studies have not only provided a clearer picture of the cellular and molecular mechanisms that cause the elimination of chromosomes in developing embryos of hybrid origin but also stimulated interest in adopting inter- and intra-specific hybridization as a practical technique to induce haploidy in different crops.

Centromeres play a vital role in the segregation of chromosomes, and they direct the assembly of the kinetochore during mitosis and meiosis. Kinetochores interact with the microtubules of the spindle, allowing chromosome migration during nuclear division. Kinetochore consists of several proteins, of which a variant of histone H3 known as CENH3, which replaces conventional H3 in centromeric nucleosomes, is known to be crucial for proper segregation of chromosomes. Thus, this centromere-specific CENH3 has been the focus of deep analysis in attempts to elucidate the mechanism of chromosome elimination following wide hybridization as well as to engineer haploid induction in plants.

One of the first studies to demonstrate that haploids could be obtained by modifying CENH3 was conducted on the model plant *Arabidopsis thaliana* [99]. CENH3 has two domains: a conserved C-terminal Histone Fold Domain (HFD) and a highly variable N-terminal tail. The modification involved replacing the hypervariable N-terminal tail domain of CENH3 with the tail of conventional H3 fused with a green fluorescent protein (GFP) tag. When *CENH3* mutants expressing altered CENH3 proteins were crossed to wild type, chromosomes from the mutant were eliminated, producing haploid progeny. Thus, they established a practical basis for engineering genome elimination through the use of altered CENH3 proteins.

Some studies that followed demonstrated that substitution of amino acids in the more conserved HFD domain can produce *A. thaliana* plants with normal fertility yet act as haploid inducers on outcrossing [100,101]. In a more recent study, Kuppu et al. (2020) [102] created clustered regularly interspaced short palindromic repeats (CRISPR)/Cas9-mediated in-frame deletions in the HFD domain of *A. thaliana*, producing mutant lines that displayed haploid induction when outcrossed. From these results, it becomes clear that modifications in both the hypervariable and conserved domains of CENH3 can give rise to mutants with haploid induction capability. Given that CENH3 is found universally in all eukaryotes, the potential of these methods as non-transgenic seed-based approaches for haploid induction in crop plant species needs to be analyzed further and exploited.

Barley was one of the first crop plants in which CENH3 activity was investigated [103]. Using the classical “bulbosum method”, they examined the role of CENH3 in the segregation of chromosomes in dividing cells of unstable hybrid embryos of *H. vulgare* × *H. bulbosum*. They observed that CENH3-positive chromosomes segregated normally, while CENH3 was lacking in the lagging chromosomes of *H. bulbosum*. Inactivity of CENH3 affected the interaction between the kinetochore of lagging chromosomes and tublinfibers, which in turn triggered distorted segregation of *H. bulbosum* chromosomes. At the end of mitosis, lagging chromosomes formed micronuclei. Due to their depleted CENH3 activity, micronucleated chromosomes were unable to participate in mitotic divisions any further, eventually leading to the complete elimination of *H. bulbosum* chromosomes in mature embryos. Expression analysis of *CENH3* during different stages of hybrid embryo development revealed that the gene is transcribed by both parental genomes. However, since the centromeres of *H. bulbosum* in unstable hybrids lacked CENH3, it was assumed that despite transcription, CENH3 was not incorporated into their centromeres, which prevented these chromosomes from undergoing normal segregation. This study helped to a great extent to unravel the molecular mechanism of uniparental chromosome elimination in interspecific barley hybrids.

**Figure 2:**
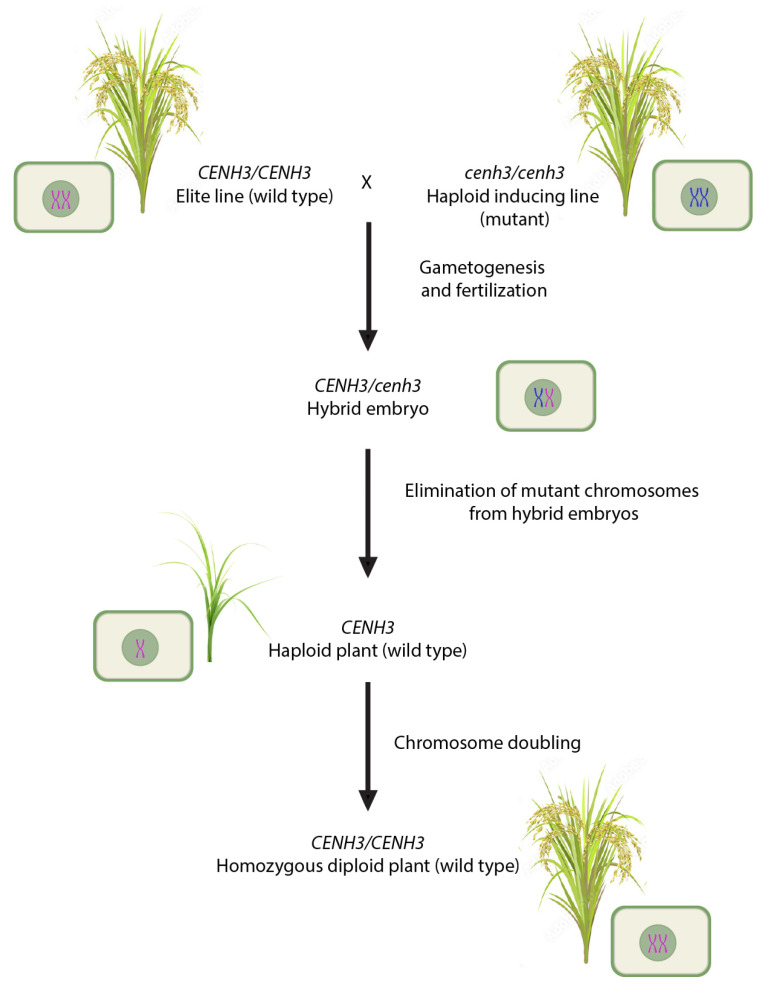
A scheme for developing *cenh3*-based haploid inducer system in rice.

In view of these new discoveries, it is useful to examine how CENH3-mediated centromeric dysfunction can be exploited for haploid induction in other cereals, particularly rice. Presently, CENH3-mediated haploid induction has been investigated mainly in the model plant *A. thaliana* [99,100,101]. As illustrated in the schematic diagram in Figure 2, the technique can be extended to any crop species provided that either naturally occurring *cenh3* mutants can be identified in its germplasm or created through gene editing. Kalinowska et al. (2019), in their review, have identified a patent describing the development of three haploid-inducing rice lines by the substitution of single amino acids in the N-terminal region of CENH3, resulting in up to 1% haploid progeny [104]. In a recent study, several CENH3 variants were identified and characterized in the genus *Oryza* [105]. In silico analysis of sequence variation at the *OsCENH3* locus identified many synonymous substitutions, while a few non-synonymous substitutions affected the protein conformation to some extent. However, it is not yet known whether this natural variation will influence haploid induction. Nevertheless, this study lays the groundwork for understanding how *OsCENH3* can be manipulated to obtain rice plants that are capable of inducing haploidy following inter- or intra-specific crosses.

### 3.2. Use of Haploid Inducer Lines

Among the cereals, maize is the only crop in which haploid inducer lines are used to produce haploids on a commercial basis at present. Success in maize is due to the presence of several natural haploid inducer lines, which have significantly increased the efficiency of developing inbred lines from cross-bred germplasm resources in maize. Haploids are produced in vivo by crossing haploid inducer lines onto non-inducer lines, resulting in a small proportion of seeds with haploid embryos. Depending on the haploid inducer line that is used in the cross, the progeny will either be paternal or maternal haploids. Subsequent treatment with chromosome-doubling agents such as colchicine yields doubled haploid plants that are completely homozygous.

#### 3.2.1. Paternal Haploids

The maize line Wisconsin-23 (W23) has been identified as a paternal haploid inducer with an approximately 3% haploid induction rate (HIR). Pure line W23 carries a spontaneous mutation in the *ig1* gene (*indeterminate gametophyte 1*) located on chromosome 3. The wild-type allele encodes the LATERAL ORGAN BOUNDARIES (LOB) domain-containing protein, which is a transcription factor responsible for the development of lateral organs in higher plants [106]. In the mutant line, the Hopscotch retrotransposon is inserted into the second exon of the gene, which is located upstream of the site encoding the LOB domain. Mutants homozygous for *ig1* are known to display a variety of phenotypes, including abnormal leaf morphology and abnormal development of the embryo sac [107]. During normal female gametogenesis, the embryo sac transits from a proliferative phase to a cellularization phase, giving rise to an embryo sac with four cell types: synergids, antipodals, egg cells, and central cells. In the *ig1* mutant, the proliferative phase is prolonged, leading to the production of extra gametes. This results in an abnormal fertilization process that produces seeds with haploid embryos of either maternal or paternal origin. However, maternal haploid frequencies are extremely low (0.1%) and depend on the non-inducer genetic background. Even though the exact mechanism of haploid induction bythe *ig1* mutant is not fully understood, the ability to produce paternal haploids using the W23 line as the female parent has been harnessed in maize breeding programs [104]. It is employed to transfer the genome of one variety of maize to the cytoplasm of another, particularly for converting inbred lines to their isogenic cytoplasmic male sterile forms [107].

The practical application of the *IG1*-system for haploid induction has not progressed much beyond its utilization in the breeding of the maize crop. Even though rice chromosomes display a high degree of synteny with maize and the rice ortholog, *OsIG1*, was shown to be involved in the regulation of the development of flower organs and the female gametophyte [108], it is yet unknown whether the *OsIG1* gene can be suitably adapted for haploid induction in rice. Down-regulation of the *OsIG1* gene was demonstrated to cause disruption in embryo sac development in rice, similar to that observed in the maize *ig1* mutant [108]. Even though these studies are encouraging, it may be premature to identify the practical application of the *OsIG1* gene for the development of haploid lines in rice as the exact mechanism of haploidization is still unknown.

#### 3.2.2. Maternal Haploids

The two widely accepted mechanisms of maternal haploid induction in maize are either parthenocarpic seed development following failure of the egg cell to be fertilized [109] or the elimination of paternal chromosomes in the zygote soon after fertilization [110].

Prigge et al. (2012) proposed that maternal haploid induction in maize was a threshold character under polygenic control [111]. Genetic mapping studies in maize have located two main QTLs affecting haploid induction potential, *quantitative haploid induction rate 1* (*qhir1*) and *quantitative haploid induction rate 8* (*qhir8*), on maize chromosomes 1 and 9, respectively [112]. The two loci, *qhir1* and *qhir8*, determine 66% and 20% of the genetic variation associated with maternal haploid induction, respectively [111,113]. Fine mapping has enabled the identification of two genes within these QTLs, *MATRILENEAL* (*MTL*) on *qhir1* and *DOMAIN OF UNKNOWN FUNCTION 679 MEMBRANE PROTEIN* (*DMP*) on *qhir8*. While the wild-type alleles of the two genes were shown to produce normal phenotypes, the mutant alleles, *mtl* and *dmp,* influenced the haploid induction phenotype. In the recent past, several studies have attempted to characterize the two genes, *MTL* and *DMP*.

In 2017, several research groups analyzed the gene located on *qhir1*, which codes for a pollen-specific patatin-like phospholipase protein. The gene was given the name *MATRILENEAL* (*MTL*) by Kelliher et al. (2017) [114], which was also called *ZmPLA1* [115] or *NOT LIKE DAD* (*NLD*) [116]. A sequence comparison between non-inducer and inducer lines of maize revealed that a mutation caused by a 4-bp insertion at the carboxy (C)-terminal of the fourth exon in this gene was primarily responsible for the haploid inducing phenotype and associated traits. The insertion resulted in a frameshift mutation with a premature stop codon, which led to a truncated protein. Kelliher et al. (2017) [114], through functional studies, showed that wild-type MTL protein was localized in the male gamete, and this sperm-specific localization was affected in *mtl* mutant lines in haploid inducer pollen. Upon further analysis of its subcellular localization, Gilles et al. (2017) [116] reported that complete wild-type protein was expressed in mature pollen membranes but was absent in pollen of the inducer lines. Irrespective of whether the *mtl* gene was coupled with an endogenous or a constitutive promoter, the truncated protein was not expressed at detectable levels. These studies indicated a role for full-length patatin-like phospholipase in normal fertilization, whereas mis-localization of the defective protein in the mutant lines was connected with the inability of the male gamete to affect fertilization of the egg cell, which led to maternal haploid induction.

The source of *mtl* mutation in the haploid inducer lines that have been developed so far in maize can be traced back to the maize line ‘Stock6′, which is a pureline of maize with a HIR of about 3% [117,118]. Intraspecific crosses of ‘Stock6′ with a haploid-inducing line possessing the *ig1* mutant allele have yielded maize lines with a higher HIR of about 10%. By using the haploid inducing lines as pollen parents in crosses with non-inducer female lines, it is possible to obtain a proportion of seeds containing haploid embryos of maternal origin.

In rice, through in silico analysis, 16 patatin-like phospholipases have been annotated [119]. Of these genes, *OspPLAIIѱ*/*PLP1,* located on rice chromosome 3 in a region that is syntenic to maize chromosome 1, is identified as the rice ortholog of *ZmMTL*. Yao et al. (2018) [120] produced knockout mutants of *OspPLAIIѱ* using the gene editing tool CRISPR/Cas9. Analysis of 14 mutants grown from selfed seed showed on average a HIR of approximately 6% in rice. Since these results were comparable to those produced by *ZmMTL* mutants, the *OspPLAIIѱ* gene was renamed *OsMTL* [120].

Following the initial work of Yao et al. (2018) [120] on rice, studies have emerged demonstrating the practical applicability of genome-edited *OsMTL* lines to develop a successful haploid induction system in rice. A protocol was streamlined by Liu et al. (2021) [121] for the development of haploid inducer lines in the *indica* rice line IR58025B by CRISPR/Cas9-mediated genome editing. The target sites for editing were exon 1 and exon 4 of the *OsMTL* gene, through which a series of mutations were created with HIR of 6% when selfed and 2–5% when out-crossed.

In a further extension of the application of *OsMTL* gene editing for practical breeding of rice, Wang et al. (2019) [122] studied the combined effect of *OsMTL* gene mutations and mutations of three other genes that are crucial for the regulation of meiotic processes. Mutations in the latter three genes lead to the development of the *MiMe* (Mitosis instead of Meiosis) phenotype, in which meiosis is replaced by a mitosis-like division, resulting in the production of male and female clonal diploid gametes. In an elite hybrid rice variety of *indica*-*japonica* dual origin, they created mutations in all three *MiMe* genes. As predicted, the triple mutant F1 hybrids produced diploid clonal gametes, suggesting that meiosis was turned into mitosis-like division consistent with the *MiMe* phenotype. However, since self-pollination of *MiMe* plants doubles the ploidy at each generation, it was necessary to trigger haploid induction in the transformants. Thus, Wang et al. (2019) [122] simultaneously edited the *OsMTL* gene and proved that, when combined with *MiMe* technology, it could be used to fix heterosis in self-pollinating rice hybrids. This has created a novel method for the propagation of hybrid rice via clonal seeds.

In another recent study, Wang et al. (2022) [123] successfully produced a large number of haploid-inducing rice lines with different heading dates using CRISPR/Cas9 editing of the *OsMTL* gene. Thus, the field is now wide open for the exploitation of this exciting new technology for haploid induction in rice.

Apart from the *MTL* gene on *qhir1*, the *DMP* gene located on *qhir8* has been implicated in maternal haploid induction in maize. The *DMP* mutation, however, is not derived from ‘Stock6’. Zhong et al. (2019) [124] observed that a single base pair substitution in the coding region of *DMP* and the consequent change in one amino acid from methionine to threonine were responsible for the higher HIR in the mutant plants. Expression analysis revealed that *DMP* has a similar expression pattern to *MTL* and is highly expressed in mature pollen, with the protein localized on the plasma membrane. While *dmp* on its own has a relatively low HIR of around 0.3% [122], it can act to enhance the haploid induction efficiency of *mtl* by five- to six-fold when both mutations are present together [125]. While *MTL* genes have been identified only in monocots, *DMP* orthologues are conserved in both monocots and dicots [114]. Table 1 summarizes information pertaining to the genes that have been identified as suitable candidates for haploid induction in plants and their potential utilization in rice.

### 3.3. CENH3 and MTL Are Two Promising Candidate Genes for Haploid Induction in Rice

While there is a growing body of research that describes the development of in vivo haploid induction methods and their utilization in various crop species, among several genes that were implicated in having a role in haploid induction, the two genes *CENH3* and *MTL* stand out for their amenability to be adopted across cereals, including rice. Their usefulness is based on functional conservation; whereas *CENH3* is conserved among most eukaryotes, *MTL* is highly conserved among monocots, making them ideal candidates for genetic manipulation to achieve predictive outcomes.

Studies conducted so far suggest that any modification of CENH3 that weakens centromere function can lead to haploid induction [126]. CENH3 consists of a relatively conserved C-terminal region and a highly variable N-terminal region. Initial studies on rice have shown that, despite its fast-evolving nature, mutations in the N-terminal tail have the potential to create haploids in both *Arabidopsis* [99] and rice [104]. It remains to be seen whether the more conserved HFD domain can be a target for mutagenesis to create haploids in rice.

The usefulness and practical application of *MTL* mutants in generating haploids in rice have already been demonstrated [120,122]. Wang et al. (2019) [122] have discussed in their review the potential application of the two genes, *MTL* and *CENH3,* for haploid induction in plants. Prospects for the development of in vivo haploid induction systems for rice were separately reviewed by Kyum et al. (2022) [96]. Their work indicates the growing interest of geneticists and plant breeders in this dynamic field.

## 4. Conclusions and Future Prospects

The presence of a reliable haploid induction system is extremely valuable for crop breeding, as it saves time and resources devoted to the development of homozygous breeding lines. Haploid plants can undergo chromosome doubling, either spontaneously or artificially, resulting in true breeding lines in a single generation. Currently, in vitro anther and microspore culture are the primary methods for haploid induction in rice. Even then, these techniques are not easily applicable to *O. sativa* varieties that possess an *indica* genetic background. The recalcitrance of the *indica* germplasm has been discussed in many previous studies [39,40,41,42]. The genotype-dependency of the two main steps in anther and microspore culture, callus induction and plant regeneration, can be circumvented by using in vivo haploid induction approaches. That is, if haploid-inducing lines are available in crop plants of interest, such lines can be used as parents in crosses with elite breeding lines to produce offspring with the haploid chromosome complement of the elite parent. A subsequent step of chromosome doubling will enable the generation of fully homozygous inbred lines from agronomically superior but tissue culture-recalcitrant genotypes.

Therefore, the future of haploid induction in *indica* rice, which is the subspecies of *O. sativa* that is least amenable to in vitro culture, may lie in the development of in vivo haploid induction systems. Until recently, there were no in vivo methods of haploid induction in rice. However, recent studies investigating *MTL* and *CENH3* genes in rice have opened up new possibilities for developing in vivo haploid induction systems. The genome editing tool CRISPR/Cas9 is currently the most viable option for creating knockout mutants of *MTL* and *CENH3* rice orthologs. Although an initial in vitro step is still required for regenerating CRISPR-edited plants with mutant alleles, transgenic protocols can be optimized for a specific tissue culture-responsive genotype. Once a haploid-inducing line is produced through CRISPR-editing, it can be used for haploid induction through intraspecific crosses with any desired cultivar, facilitating a convenient system for haploid plant production.

Additionally, it has been argued that the progeny of CRISPR/Cas9-edited haploid-inducing parents should be exempt from GMO classification as they do not possess the altered genomes [127]. Since the haploid progeny will consist only of chromosomes derived from the non-inducer parent, concerns that are currently associated with genome-edited crops will have no bearing on crops produced through this technique.

## Figures and Tables

**Table 1:** Gene targets for in vivo haploid induction in plants, with potential application in rice.

Gene	Wild Type (WT) Gene Product	WT Expression Analysis	Mutagenic Sites	Mutant Phenotype	Rice Ortholog/Potential for Rice Haploid Induction
*CENH3*	Centromere-specific CENH3 protein (histone variant) consisting of two domains: conserved C-terminal HFD and highly variable N-terminal tail	Kinetochore-loading of CENH3 protein leading to precise chromosome segregation during cell division.	Both domains are targets.	Impaired chromosome segregation during cell division and uniparental chromosome elimination in young embryos.	*OsCENH3*/ Limited studies in rice have shown an HIR of approximately 1% [104].
*IG-1*	LOB-domain protein (Transcription Factor)	Normal female gametogenesis leading to an 8-celled embryo sac (and the development of lateral organs).	Second exon of the gene, upstream of the encoded LOB domain.	Delayed cellularization of the embryo sac leading to extra gametes and abnormal fertilization.	*OsIGI*/No reports of haploid induction in rice.
*MTL*	Pollen-specific patatin-like phospholipase protein	Localization of proteins in mature pollen membranes leads to normal fertilization of the egg cell.	Exons 1 and 4 (CRISPR/Cas 9 knock-outs)	Mis-localization of the defective protein affects normal fertilization leading to maternal haploid induction.	*OsMTL*/ HIR of 6% when selfed and 2–5% when out-crossed in rice [120].
*DMP*	Domain of unknown function 679 Membrane Protein	Expressed in mature pollen and involved in gamete fusion (similar to *MTL*)	Coding region	Not clearly defined	*OsDMP*/No reports of haploid induction in rice

## Data Availability

Not applicable to this article.
